# Tetramethyl Bisphenol F: Organ- and System-Specific Toxicity, Current Status, and Perspectives

**DOI:** 10.3390/ijms26199280

**Published:** 2025-09-23

**Authors:** Inho Hwang, Xiang-Shun Cui, Eui-Bae Jeung

**Affiliations:** 1 Laboratory of Veterinary Biochemistry and Molecular Biology, College of Veterinary Medicine, Chungbuk National University, Cheongju 28644, Chungbuk, Republic of Korea; darkpower777@nate.com; 2 Department of Animal Science, Chungbuk National University, Cheongju 28644, Chungbuk, Republic of Korea; xscui@chungbuk.ac.kr

**Keywords:** TMBPF (tetramethyl bisphenol F), endocrine-disrupting chemicals (EDCs), BPA alternatives, toxicological profile, food contact materials

## Abstract

Tetramethyl bisphenol F (TMBPF) is being increasingly used as a Bisphenol A (BPA) substitute, particularly as a coating material for food and beverage cans. Unlike BPA, TMBPF is considered safe because of the lack of reported estrogenic effects, and it is often marketed under the “BPA-free” label. Initial cell-based assays and rat toxicity studies indicated much lower systemic and sex hormone-related toxicity of TMBPF compared with BPA, which has facilitated its substitution and significant market expansion. Since 2021, however, a growing body of research has reported various adverse effects of TMBPF across multiple biological systems. These include cytotoxicity associated with apoptosis and endocrine-disrupting effects on the thyroid axis, skeletal system, neurodevelopment, and reproductive function. Although the effects on the estrogen and androgen systems, as well as obesogenic potential, show variability across studies, several studies have indicated significant biological impacts. Of particular concern is the potential neurodevelopmental toxicity, which may manifest only after long-term exposure and is often irreversible. Even if current leaching levels from food contact materials are minimal, environmental accumulation and biomagnification over time may pose significant risks. Therefore, comprehensive toxicological profiling of TMBPF is essential. This review summarizes the current toxicological findings on TMBPF and discusses the implications for future research and regulatory considerations, highlighting the importance of early attention to potential public health impacts. Strengthening the toxicological evidence base will help inform regulatory frameworks and support proactive measures to safeguard consumer safety as the use of TMBPF expands.

## 1. Introduction

### 1.1. Background

Bisphenol A (BPA) has been widely used in coatings, plasticizers, epoxy resins, and flame retardants. It has been shown to act as an endocrine disruptor and exert adverse effects on the reproductive system, as well as on metabolism and neurodevelopment. As a result, BPA has been classified as an endocrine-disrupting chemical (EDC), and regulations on its use have become increasingly stringent [1]. Consequently, it has been replaced by alternative substances depending on the application. These alternatives include other bisphenol analogues with similar structures and properties, such as bisphenol F (BPF, CAS 620-92-8), bisphenol S (BPS, CAS 80-09-1), and halogenated and non-halogenated bisphenol derivatives [2]. In addition, non-bisphenol-based alternatives, such as polypropylene (PP, CAS 9003-07-0), polyethylene (PE, CAS 9002-88-4), lignin (CAS 8068-05-1), polyethersulfone (PES, CAS 25608-63-3), and polyphenylsulfone (PPSU, CAS 25667-42-9), have also been adopted for industrial applications (Table 1).

### 1.2. Tetramethyl Bisphenol F (TMBPF)

#### 1.2.1. Adoption of TMBPF as an Alternative to BPA

TMBPF is a non-halogenated bisphenol derivative structurally characterized by the presence of four methyl groups on the bisphenol F backbone (Table S1) [12]. Although this compound had been previously known, information regarding its industrial use was limited. TMBPF was identified through the in silico screening of bisphenol analogues by scientists at Sherwin-Williams, a major paint and manufacturing company, as part of their effort to develop a bisphenol-based compound without endocrine-disrupting properties [13]. Based on their internal studies and collaborations with external research institutions, Sherwin-Williams reported that TMBPF lacks estrogenic activity, has been reported to be non-genotoxic, and has very low toxicity at minimal exposure levels, which they considered sufficient to position it as a potential BPA substitute [13,14]. The company has since commercialized TMBPF as the core component of the valPure V70 system, a coating material for the internal lining of food cans. 

#### 1.2.2. Early Toxicity Studies of TMBPF

Soto et al. (2017) conducted various in vitro and in vivo assays to assess the endocrine-disrupting potential of TMBPF [15]. The results from in vitro assays, including the E-SCREEN bioassay and the estrogen receptor transactivation assay, suggested that TMBPF does not exhibit estrogenic activity at the cellular level, nor does it act as an agonist or antagonist of the estrogen receptor. Furthermore, no significant effects on sexual maturation endpoints have been observed in animal models. The amount of TMBPF migrating from polymer-based coatings was reported to be below the limit of quantification, suggesting that human exposure and associated risk are negligible [15]. Similarly, Szafran et al. evaluated TMBPF and its derivatives using multiple cellular models [16]. Although BPA has clear estrogenic activity, TMBPF and its analogues showed minimal to no estrogenic or androgenic activity. Interestingly, the study also reported that TMBPF has anti-estrogenic and anti-androgenic effects. Together, these investigations applied a range of mechanistic approaches to support the relative safety of TMBPF as a potential commercial alternative to BPA.

A toxicity study published in 2020 reported that TMBPF exhibits no aromatase inhibition and only weak estrogenic (E2) and testosterone-inducing effects [17]. In addition, the compound showed no androgenic effects in the Hershberger assay, with anti-androgenic effects observed only at a high dose of 1000 mg/kg/day. In a 90-day repeated-dose toxicity study, the no-observed-adverse-effect level (NOAEL) was determined to be 750 mg/kg/day in female rats and 1000 mg/kg/day in male rats. Accordingly, the authors concluded that TMBPF displays significantly lower toxicity than other bisphenol analogues.

#### 1.2.3. Expanding Toxicological Evidence and the Need for Reassessment of TMBPF

Since the publication of a study by Harnett et al. in 2021, concerns regarding the potential toxicity of TMBPF have continued to emerge [18]. Recent studies have reported possible effects on thyroid hormone signaling, skeletal development, adipogenesis, neurotoxicity, and sex hormone-related activity, even though earlier studies suggested that such endocrine-disrupting effects were minimal. EDCs can accumulate in the human body through direct exposure to products and indirect routes such as environmental contamination and bioaccumulation in the food chain [19]. Although indirect exposure may take time to manifest, direct ingestion through oral contact with food or beverage container linings can result in more rapid and higher internal exposure [20]. Although several studies consistently reported negligible migration of TMBPF from food-contact coatings, EDC-related toxicity may develop cumulatively over prolonged periods [21,22]. Therefore, considering the evidence of endocrine-related effects, more focused research and regulatory evaluation are warranted.

### 1.3. Objectives and Structure of the Study

TMBPF has increasingly been adopted as a substitute for BPA, making it a chemical of growing relevance in daily life and a potential source of human exposure. Numerous toxicological studies have been published, but a comprehensive overview of these findings is still lacking. The present review aims to systematically examine and organize the available toxicological evidence on TMBPF according to organ and system-specific outcomes, identify characteristic features of its toxicity, and provide insights for future research directions. In addition, this review considers current market trends and regulatory contexts to offer perspectives on how TMBPF can be more safely managed, thereby informing both scientific evaluation and policy-making.

## 2. Organ System-Based Toxicological Evidence of TMBPF

As the toxicity of BPA became widely recognized and regulations tightened, the need to identify suitable industrial alternatives became increasingly important. Consequently, various substitutes began to be used even before sufficient toxicological data accumulated. TMBPF, one of the representative BPA alternatives, has since been assessed for a range of potential toxic effects, either alone or in comparison with other bisphenol analogues, across various biological models. This review summarizes the literature based on the types of toxicities reported.

### 2.1. Apoptotic and Cytotoxic Effects

Harnett et al. conducted a comparative study evaluating the cytotoxicity and apoptosis-inducing effects of three BPA substitutes (BPS, BPAF, and TMBPF) in rat and human cell models, using BPA as a reference compound [18]. Their results showed that TMBPF exhibited a bimodal toxicity pattern, with potent effects observed at low and high concentrations. The LC_50_ values were reported as 0.88 μM in adult female rat adipose-derived stem cells (rASCs) and 0.06 μM in human mesenchymal stem cells (hMSCs), which were significantly lower than those of BPA (1 μM and 10 μM for rASCs and hMSCs, respectively). An increase in the activated caspase-6 levels was also observed, suggesting that the observed cell death occurred through caspase-mediated apoptotic pathways. In a follow-up study, the authors provided additional supplemental data to strengthen the reliability of their results. Their findings indicated that TMBPF may exhibit greater toxicity than BPA. This study was the first to show the cytotoxic potential of TMBPF, underscoring the need for broader toxicological investigations into its safety profile.

### 2.2. Estrogenic Activity and Female Reproductive Effects

As mentioned in the Introduction, the potential estrogenic activity of TMBPF was one of the earliest toxicological endpoints to be investigated. Because BPA, the parent compound, acts on the estrogen receptors, any suitable substitute was expected to exhibit minimal or no such activity. Early studies suggested that TMBPF had negligible estrogenic and reproductive effects, which contributed to its industrial adoption as a safer alternative [15,16].

A recent study by Fan et al. (2025) challenged this assumption by showing that TMBPF may disrupt endogenous estrogen homeostasis and adversely affect female mouse reproductive health [23]. The researchers examined the effects of TMBPF using pregnant CD-1 mice and human granulosa cells (KGN). Pregnant mice were administered TMBPF from gestational day 7 to postnatal day 21. The serum levels of estradiol (E2), follicle-stimulating hormone (FSH), and luteinizing hormone (LH) were elevated in F1 female offspring. In addition, a dose-dependent decrease in ovarian organ indices and histological evidence of ovarian fibrosis were observed. These findings were interpreted as indicative of ovarian damage resulting from maternal TMBPF exposure. In KGN cells, a 48 h TMBPF treatment led to a significant decrease in cell viability, as confirmed by viability assays, flow cytometry analysis, and 5-ethynyl-2′-deoxyuridine (EdU) staining, a marker of newly synthesized DNA. Mechanistic studies revealed increased oxidative stress, mitochondrial dysfunction, and G2/M phase arrest. Protein expression analysis showed significant increases in p53, p21, and γ-H2AX, along with decreased CDK1 levels. Senescence-associated β-galactosidase staining and transcriptomic profiling confirmed that this process was mediated via the ERRβ–p21 signaling pathway. This study was the first to show that TMBPF may exert transgenerational toxicity on the female reproductive system. These findings raise concerns that TMBPF, which was previously considered safe in terms of the estrogen-related effects, may pose significant endocrine risks.

### 2.3. Androgenic Activity and Effects on the Male Reproductive System

Similar to the findings on estrogenic activity, previous studies reported that TMBPF had no significant androgenic or anti-androgenic effects. Despite this, more recent investigations suggested that this assumption may not hold. Park et al. reported that TMBPF exhibits notable anti-androgenic activity [24]. Using a yeast-based androgen receptor (AR) reporter assay, the researchers evaluated the androgenic and anti-androgenic potential of BPA and its analogues, including BPS, 4PP, TMBPF, and TMBPA. Molecular docking and molecular dynamics simulations were conducted to assess the interaction with the human AR ligand-binding domain (AR-LBD). TMBPF showed no androgenic activity but displayed relatively strong anti-androgenic activity, with a potency approximately 57.2 times higher than that of BPA. This trend is consistent with previous reports [16]. Molecular docking analysis showed that TMBPF interacts with the key residues in AR-LBD, particularly Gln711 and Thr877.

Higley et al. investigated the effects of BPA, BPS, and TMBPF on male fertility using *Caenorhabditis elegans* [25,26]. Exposure to 0.5 mM TMBPF for 48 h resulted in a decrease in sperm size and embryo viability. Unlike BPA, TMBPF did not significantly reduce the sperm activity or offspring size, suggesting that its overall toxicity in this model was less severe. Although this study was conducted in an invertebrate species and lacks mechanistic detail, it is the first to report the male reproductive effects of TMBPF. These findings suggest that TMBPF may have anti-androgenic effects, primarily in the male reproductive systems. Nevertheless, considering the limited number of studies, more detailed and focused research is warranted to clarify its potential risks.

### 2.4. Thyroid Hormone Disruption

One of the representative mechanisms of EDCs is interference with the thyroid hormone system. These chemicals may disrupt thyroid hormone homeostasis by affecting hormone synthesis, transport, metabolism, or receptor signaling, which may result in thyroid hormone imbalances resembling hyperthyroid- or hypothyroid-like conditions [27]. For BPA, several studies have confirmed such mechanisms. BPA antagonizes thyroid hormone receptors (TRs) and suppresses T3-dependent gene transcription. It has also been shown to alter the expression and enzymatic activity of the genes involved in thyroid hormone synthesis, such as thyroperoxidase (*Tpo*), the sodium/iodide symporter (*Nis*), and thyroglobulin (*Tg*) [28]. Animal studies showed that BPA exposure affects the serum levels of T3, T4, and TSH, with early developmental exposure linked to impaired brain development and behavioral alterations [29,30]. Furthermore, epidemiological studies in humans have found correlations between BPA concentrations in urine or serum and thyroid hormone indices (TSH, T3, T4). Several birth cohort studies have suggested that maternal BPA exposure can affect neonatal thyroid function [31]. Overall, BPA is widely recognized as a thyroid-disrupting endocrine chemical based on the evidence from cellular, animal, and human studies.

In contrast, studies on the thyroid-disrupting potential of TMBPF are limited. On the other hand, Kim et al. (2022) reported that zebrafish exposed to TMBPF for 14 days showed a significant increase in whole-body T3 levels and a decrease in growth hormone (GH) expression [12]. This was accompanied by the increased rates of embryonic coagulation and malformation, reduced larval body weight, delayed growth, and decreased hatchability and survival. The authors also observed significant changes in the expression of the genes related to the hypothalamus–pituitary–thyroid (HPT) axis and the growth hormone/insulin-like growth factor (GH/IGF) axis. Hence, TMBPF may disrupt thyroid hormone synthesis or metabolism, potentially inducing a hyperthyroid-like state and triggering compensatory disturbances in the GH/IGF signaling pathway. Although based on a single non-mammalian model, this study provides initial evidence of thyroid-disrupting potential for TMBPF and highlights the need for further mechanistic and mammalian studies similar to those conducted for BPA.

### 2.5. Obesogenic Effects

Obesity is one of the most prevalent and serious health concerns in modern society [32]. Although its etiology is multifactorial, chemicals that can promote obesity through endocrine disruption are classified as “obesogens.” These substances may interfere with hormonal regulation, promote adipocyte differentiation, impair lipid metabolism, alter appetite, and disrupt energy homeostasis [33]. BPA and several other EDCs have been categorized as obesogens [34].

Regarding TMBPF, two studies have reported contrasting outcomes on its potential obesogenic effects. Cohen et al. (2021) assessed the effects of BPA, BPAF, and TMBPF on adipogenesis and lipid accumulation in hMSCs [35]. Unlike the other bisphenol analogues, which promoted adipocyte differentiation and lipid accumulation, TMBPF suppressed adipocyte development. In particular, even at very low concentrations, TMBPF exhibited clear anti-adipogenic activity along with pronounced cytotoxicity and apoptosis-inducing effects. Although the same research group reported that TMBPF was approximately 100 times more cytotoxic than BPA, they suggested that its anti-adipogenic effect could not be explained by cytotoxicity alone and called for further investigation.

In contrast, Singh et al. (2024) reported opposing findings [36]. Using murine 3T3-L1 preadipocytes, the authors evaluated the adipogenic effects of BPA and its analogues, including BPF, BPAP, and TMBPF. Among these, TMBPF exhibited the strongest adipogenic response, significantly enhancing lipid accumulation and upregulating the expression of the key adipogenic markers such as lipoprotein lipase (*Lpl*), fatty acid-binding protein 4 (*Fabp4*), and perilipin (*Plin*). Based on these results, the authors concluded that TMBPF acts as a PPARγ agonist and may function as an obesogenic compound and a metabolic disruptor.

The opposing results between the two studies may largely reflect differences in experimental conditions. Cohen et al. (2021) used human adipose-derived stem cells and applied relatively low concentrations (0.01–0.1 µM) during an 11-day differentiation protocol, whereas Singh et al. (2024) employed murine 3T3-L1 preadipocytes exposed to higher concentrations (1–20 µM) during an 8-day differentiation period [35,36]. Such variations in concentration ranges and cell type may have contributed to the divergent outcomes, with stress responses being more pronounced under the former conditions, while adipogenic differentiation predominated under the latter.

Although these findings seem inconsistent, they consistently suggest that TMBPF influences adipocyte-related pathways, warranting further investigation. Therefore, further studies are needed to determine the precise conditions and mechanisms under which TMBPF influences adipogenesis.

### 2.6. Skeletal Effects

Among the reported toxicological effects of TMBPF, its impact on bone metabolism has also been reported. In a 2021 study, Kim et al. examined the effects of BPA, BPF, and TMBPF on osteoclast differentiation [37]. Using the mouse macrophage cell line RAW264.7, they induced differentiation into osteoclasts and treated the cells with each compound for three days. Tartrate-resistant acid phosphatase (TRAP) staining was performed to assess the osteoclast activity, and mechanistic studies were conducted using real-time PCR and Western blotting. The results showed that TMBPF led to the most pronounced increase in osteoclast area and the number of multinucleated osteoclasts compared to BPA and BPF. This was accompanied by the upregulation of osteoclast-related genes such as nuclear factor of activated T-cells cytoplasmic 1 (*Nfatc*1), *Acp5* (encoding TRAP), and cathepsin K (*Ctsk*). In addition, TMBPF enhanced the expression of proteins in the mitogen-activated protein kinase (MAPK) pathway, including the phosphorylation of c-Jun N-terminal kinase (JNK) and p38. It is well established that the MAPK pathway is a central signaling cascade regulating cell growth, differentiation, and apoptosis, and within osteoclastogenesis, p38 MAPK and JNK play particularly critical roles [38]. Activation of p38 promotes *Nfatc1* expression and thereby induces osteoclast-specific genes such as *Acp5* and *Ctsk*, while JNK stimulates c-Jun/AP-1 activity, which cooperates with *Nfatc1* to drive osteoclast differentiation and function [39]. Excessive activation of these MAPK signaling events can in turn amplify bone resorption and disrupt skeletal homeostasis. Integrating these findings with the mechanistic background, the authors concluded that TMBPF may significantly influence bone remodeling [37]. Although this conclusion is based on a single study, enhanced osteoclast activity is associated with bone-related disorders such as osteoporosis, osteomalacia, and hypercalcemia [40]. The significantly stronger effects of TMBPF compared to other bisphenol analogues highlight the need for further study into its skeletal toxicity.

### 2.7. Developmental Toxicity

#### 2.7.1. Teratogenic and General Developmental Effects

EDCs, when introduced during pregnancy, may have developmental and teratogenic effects in the fetus. BPA has been widely studied in this context and has exhibited teratogenic potential across various models [41]. Harnett et al. evaluated TMBPF for its developmental toxicity and teratogenicity [14]. The researchers exposed chick embryos to BPA, BPS, BPAF, and TMBPF between embryonic days 5 and 12, and assessed the dose-dependent morphological outcomes. The results showed that most bisphenol compounds significantly disrupted embryonic growth, development, and survival. Embryos exposed to these substances exhibited a reduced body size and weight, approximately half that of controls. The observed malformations included craniofacial deformities, gastrointestinal anomalies, and irregular body pigmentation. Mortality was also increased markedly. Among the tested compounds, BPAF showed the highest toxicity, followed by TMBPF. BPA exhibited the lowest toxicity in that study. The reported LC_50_ values were 2.92 μM and 1.18 μM for BPA and TMBPF, respectively. Although mechanistic analyses were not conducted in this study, the authors suggested that the observed teratogenic effects may be mediated by excessive apoptosis and mitochondrial toxicity. They noted that bisphenol analogs can accumulate within mitochondria due to their lipophilic nature, triggering caspase-dependent cell death pathways and disrupting neural and craniofacial development [18,35,42,43]. Although this study presented only a single dataset and had limitations, such as the use of a non-mammalian species, lack of mechanistic insight, and direct dosing at relatively high concentrations, it highlighted the potential of TMBPF to have strong developmental toxicity. Its relatively high toxicity compared with other bisphenol analogues highlights the need for further studies in physiologically relevant models.

#### 2.7.2. Neurodevelopmental Toxicity

In recent years, increasing attention has been given to the neurodevelopmental toxicity of EDCs, particularly concerning neurodevelopmental disorders such as autism spectrum disorder (ASD) and attention deficit hyperactivity disorder (ADHD) [44,45]. Neurodevelopmental toxicity can present acutely, leading to lethality, but more often arises as chronic effects after prolonged exposure during fetal and early postnatal development [46]. These chronic effects may not be externally visible but can manifest through behavioral abnormalities [47].

Liang et al. (2023) conducted a notable study on the neurodevelopmental toxicity of TMBPF using a zebrafish model [48]. Zebrafish embryos were exposed to TMBPF at various concentrations starting at four hours post-fertilization (hpf) for approximately six days. At higher concentrations, the researchers observed significant embryonic lethality and reduced hatching rates. Developmental abnormalities included reduced body length, underdeveloped eyes and swim bladders, spinal curvature, and pericardial edema. Behavioral assays revealed a dose-dependent decrease in swimming speed and distance, along with reduced responses to light–dark transitions, suggesting neurobehavioral impairment. Further analysis using fluorescent labeling showed impaired development of the central nervous system (CNS), spinal motor neurons, and dopaminergic neurons. Moreover, increased production of reactive oxygen species (ROS) and elevated enzymatic activities of catalase (CAT), and superoxide dismutase (SOD) were observed, while the expression of antioxidant genes (*sod* and *cat*) was downregulated. Previous research on BPA and its analogues has similarly demonstrated that ROS accumulation disrupts mitochondrial function and calcium homeostasis. Such disturbances impair neuronal differentiation, synaptic signaling, and dopamine system development [49,50]. Importantly, co-treatment with the antioxidant N-acetylcysteine (NAC) substantially mitigated most of these toxic effects, suggesting that oxidative stress plays a central role in TMBPF-induced neurodevelopmental toxicity [48]. In addition, neurodevelopment-related genes (*synapsin IIa*, *glial fibrillary acidic protein*, and *myelin basic protein*) were downregulated, while the genes related to dopaminergic neurons (*tyrosine hydroxylase 1; th 1/2* and *dopamine transporter; dat*) were upregulated [48]. According to the authors, these transcriptional changes suggest that TMBPF impairs synaptic formation, glial support, and myelination, while also disrupting catecholaminergic systems. Th is the rate-limiting enzyme in the synthesis of both dopamine and norepinephrine, and alterations in its expression, together with changes in DAT activity, can lead to neurotransmitter imbalance, impaired synaptic transmission, and locomotor dysfunction. Although this finding is currently based on a single study using a non-mammalian model, the zebrafish is widely recognized as a reliable model for neurotoxicity screening [51]. The dose-dependent responses, mechanistic insights, and toxicity reversal through antioxidant intervention provide compelling evidence that TMBPF may pose neurodevelopmental risks through oxidative stress-related pathways.

### 2.8. Other Toxicological Studies

#### 2.8.1. Identification and Toxicological Assessment of NIAS (Non-Intentionally Added Substances)

NIAS refer to chemical compounds unintentionally present in food packaging or food contact materials [52]. These substances may arise as by-products of polymerization, degradation products, contaminants, or residual chemicals. Moreover, although their toxicity remains largely uncharacterized, they pose potential risks to consumer health. Mallen et al. (2023) analyzed NIAS potentially migrating from V70, an epoxy coating used in metal cans containing TMBPF-DGE [53]. They constructed a database of candidate NIAS and conducted migration testing, compound characterization, and prioritization. Seven compounds containing oxirane functionalities, which are potentially genotoxic, were identified, with TMBPF-DGE combined with hydroquinone accounting for 55% of the migration profile. This compound was selected for further toxicological evaluation. The estimated daily intake (EDI) was calculated as 5.2 μg/person/day. In vitro genotoxicity assays, including the micronucleus test and the Ames test, had no genotoxic effects. Although Sherwin-Williams, the manufacturer of V70, conducted the study, it followed the European Union and EFSA guidelines for NIAS analysis. Hence, its findings contribute to the toxicological evaluation of food contact materials.

#### 2.8.2. Effects on Gut Microbiota

Beyond digestion and nutrient absorption, the gastrointestinal tract plays a central role in host metabolism, immune function, and even brain development via the gut–brain axis [54]. One of the key regulators of this system is the composition of the gut microbiota. Dysbiosis, or disruption of microbial balance, has been linked to various health conditions, including neurodevelopmental disorders [55]. Średnicka et al. (2024) examined the impact of BPA and its analogues, including BPS, BPF, and TMBPF, on the gut microbiota derived from healthy human fecal samples [56]. Each compound was administered at 1 mM for 48 h. The researchers analyzed the microbial diversity, community structure, short-chain fatty acid (SCFA) production, lysogenic metabolism, estrogenic activity, and the effects of the post-exposure media on Caco-2 intestinal epithelial cell viability. Among the compounds tested, TMBPF had the least effect on microbial diversity and SCFA production. Interestingly, microbial binding to TMBPF was the highest, and the bound fraction showed significantly reduced estrogenic activity. Nevertheless, the post-treatment media from TMBPF-exposed microbiota led to reduced viability in Caco-2 cells. SCFAs are known to alleviate inflammation, support tight junction integrity, and play important roles in maintaining gut health, and they are therefore often used as monitoring indicators [57,58,59]. However, their levels do not always correlate directly with intestinal toxicity. In the referenced study, BPA, BPF, and TMBPF affected α-diversity and SCFA production in the same order as their impact on Caco-2 cell viability [56]. In contrast, BPS also altered diversity and SCFA levels but did not induce cytotoxicity. The authors emphasized that microbial metabolites, rather than SCFAs themselves, may be the main contributors to cytotoxicity. This study suggests that TMBPF may adversely affect gut health, and to more clearly elucidate the underlying causes and mechanisms, further research is needed under long-term and chronic exposure conditions. Such research would benefit from employing normal cell models and adopting diverse approaches to comprehensively evaluate gut microbiota composition, SCFA production, direct toxicity, and metabolite-related effects.

## 3. Discussion

### 3.1. Characteristics of Reported Toxicity of TMBPF

Although the first toxicological studies reporting the adverse effects of TMBPF were published less than five years ago, numerous toxicities across multiple organ systems have since been identified (Table 2). These findings would suggest the need for increasing regulatory restrictions or caution regarding TMBPF. However, the opposite trend has been observed. TMBPF is being formally approved or exempted by regulatory authorities around the world. For example, in 2022, the Netherlands officially approved the use of TMBPF-DGE in metal food can coatings [60]. Washington State banned BPA, BPS, and BPF in beverage can coatings in 2023 but explicitly exempted TMBPF-based coatings [60]. Similarly, California’s proposed AB 1148 legislation to ban bisphenols in food packaging by 2025 also exempted TMBPF [61]. In the United Kingdom, the Food Standards Agency (FSA) and Food Standards Scotland (FSS) recently assessed the safety of TMBPF-DGE and concluded in 2024 that, under intended use conditions, no safety concerns were identified [60]. China has also approved certain TMBPF-DGE–containing reaction products for use in food-contact coatings, with restrictions such as a maximum use level of 20% in coating formulations, specific migration limits (e.g., 0.2 mg/kg for TMBPF-DGE and related derivatives), and prohibitions on applications for infant products or fatty foods [62]. These examples illustrate that authorizations for TMBPF-based materials are gradually expanding across different regions. The market for TMBPF continues to grow. According to Market.us, the global market value of TMBPF is projected to increase from USD 3.18 million in 2024 to USD 5.33 million by 2034, reflecting a compound annual growth rate (CAGR) of 5.3% over the next decade [63].

### 3.2. Scientific Limitations and Regulatory Gaps

These trends suggest that current research findings have not reached the threshold required for regulatory concern. TMBPF is one of the most actively used BPA alternatives, but its widespread use only began within the past decade [35]. Consequently, toxicological data remain limited. Most reported toxicities are derived from single studies, often using cell-based or alternative models. Although alternative methods are increasingly employed to reduce animal testing, such limited findings are insufficient to support regulatory decisions, and the absence of immediate evidence does not imply safety. The continued reports of toxic effects for a widely used substance highlight the need for comprehensive studies across each toxicity category. Coordinated efforts among regulatory agencies and stakeholders are also required to ensure transparent and independent safety evaluations that protect consumers from potential long-term risks.

### 3.3. Beyond BPA-Free: Uncertainties Associated with Substitute Chemicals

Although BPA is often perceived as a highly hazardous substance because of its extensive toxicological profile, it is also one of the most thoroughly studied and tightly regulated [64]. The shift toward BPA-free products reflects public awareness and market pressure [65]. Nevertheless, the risk of BPA is now managed through dynamic, evidence-based regulation. In contrast, substitute chemicals like TMBPF are often adopted before their safety profiles are fully understood. As a result, some substitutes were later found to pose greater toxicological concerns than the compounds they replaced [18]. Similarly, TMBPF has also been reported to exhibit comparable or even stronger toxicity than BPA under certain conditions (Table 3).

If current patterns persist, the concept of ‘BPA-free’ may need to be reconsidered. Thus far, there have been no confirmed reports of acute toxic effects from direct TMBPF exposure. Although low exposure levels may help mitigate short-term risks, the situation could change dramatically once TMBPF accumulates in the environment. Environmental contamination and bioaccumulation often take years to detect, but once identified, they are difficult to reverse [67]. Regulatory agencies must be proactive in evaluating existing chemicals and their substitutes and replacement products. In particular, early profiling is essential for long-term and subtle toxicities, such as neurodevelopmental toxicity. Although estimating toxicity based on current average human exposure is important, additional assessments of high-dose effects, environmental transport and degradation, and bioaccumulation potential are needed. Regulatory thresholds are recommended to be established and revised promptly in response to emerging scientific data.

## 4. Conclusions

**Figure 1:**
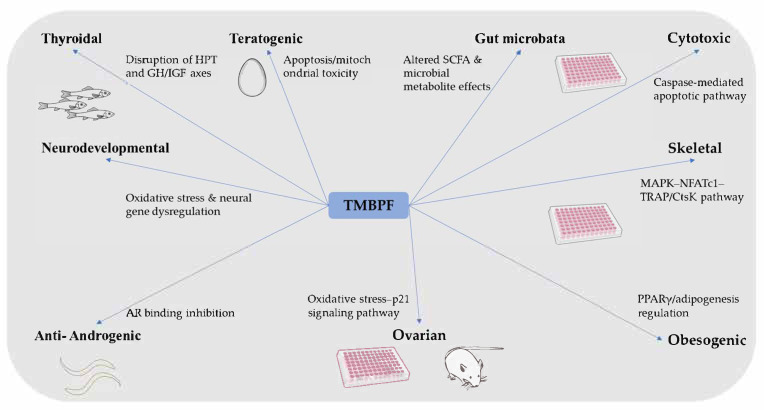
Overview of the reported toxicological effects and proposed mechanisms of TMBPF.

Recent studies suggest that TMBPF may exert diverse toxicological effects, including cytotoxicity, endocrine disruption of thyroid and reproductive axes, skeletal alterations, and possible neurodevelopmental impacts (Figure 1). Some findings indicate that, under specific experimental conditions, TMBPF can show toxicity comparable to or greater than that of BPA, despite its widespread use as a “BPA-free” substitute. Nevertheless, the overall evidence base is still limited, with many endpoints supported by only a single study and some areas, such as obesogenic potential and gut microbiota interactions, yielding inconsistent outcomes. These gaps highlight the difficulty of drawing firm conclusions about human and environmental risks at this stage. Future research would benefit from more comprehensive in vivo studies, mechanistic analyses across multiple organ systems, and long-term exposure assessments to clarify current uncertainties. At the same time, regulatory frameworks could adopt a cautious approach by incorporating early hazard profiling and updating thresholds as new data emerge. Such efforts will help ensure that substitutes like TMBPF are evaluated thoroughly, thereby supporting both public health and environmental safety.

## Figures and Tables

**Table 1:** Applications of BPA and current alternatives in industrial use.

Application Area	Traditional BPA Uses	Current Alternative Materials
Food and beverage packaging [3]	Epoxy-based can coatings, polycarbonate containers	TMBPF-based epoxy (e.g., valPure V70), polyester ^1^ resins, acrylic ^2^ resins
Baby bottles and infant products [4]	Polycarbonate plastics	Tritan™ copolyester, polypropylene (PP) ^3^, polyethylene (PE) ^4^, glass, silicone ^5^
Thermal paper receipts [5]	BPA-based thermal developing agents	Bisphenol S (BPS) ^6^, Bisphenol F (BPF) ^7^, Pergafast^®^ 2018
Medical equipment and devices [6]	Polycarbonate medical components, dental composites	BPS, BPF, ceramic fillings, and glass ionomer fillings
Electronic components [7]	Flame-retardant polycarbonates, epoxy resins for electronic boards	DOPO ^8^ (9,10-Dihydro-9-oxa-10-phosphaphenanthrene-10-oxide), Organophosphates, Metal hydroxides, Lignin-based flame retardants
Automotive and transportation, Construction materials [8,9,10,11]	Polycarbonate vehicle parts, epoxy-based adhesives, protective coatings	Lignin ^9^, Cashew nutshell liquid (CNSL) ^10^, Isosorbide ^11^, Resorcinol diglycidyl ether (RE) ^12^

as number: ^1.^ 113669-95-7, ^2.^ 94188-59-7, ^3.^ 9003-07-0, ^4.^ 9002-88-4, ^5.^ 7440-21-3, ^6.^ 80-09-1, ^7.^ 620-92-8, ^8.^ 35948-25-5, ^9.^ 9005-53-2, ^10.^ 8007-24-7, ^11.^ 16051-77-7, ^12.^ 101-90-6.

**Table 2:** Summary of published toxicity studies on TMBPF.

Toxicity	Test System	Chemical Treatment	Main Findings	Reference
Systemic	SD Rats (8 weeks)	100–1000 mg/kg/day (male), 100–750 mg/kg/day (female) for 90 days, followed by 28 days of recovery	Repeated dose 90-day oral toxicity study in rats following OECD TG 408, with NOAEL of 750 mg/kg-bw/day for females and 1000 mg/kg-bw/day for males.	Maffini et al., 2020 [17]
Cytotoxic	rASCs and hMSCs	0.01–50 uM (0.01% ethanol), 20–300 min	Strong cytotoxicity at low and high doses; LC_50_ 0.88 µM in rASCs and 0.06 µM in hMSCs; caspase-6 activation; overall more toxic than BPA.	Harnett et al., 2021 [18]
Estrogenic	LC-TOF-MS and LC-MS/MS (negative-mode ESI)	Migration extracts of TMBPF-based polymeric coating	TMBPF migration was detected below the method’s reporting limit (~0.01–0.06 ppb) following extraction in food simulants, indicating negligible migration into food.	Soto et al., 2017 [15]
	MCF7 cells (ER transactivated)	0.1–2.5 μM for 19–24 h (Transactivation Assay) 10 pM–100 μM for 6 d (E-SCREEN Bioassay)	No estrogen agonist or antagonist activity observed in the OECD TG 455 stably transfected transactivation assay, and no estrogenic activity detected in the E-SCREEN assay.	
	SD rats (female)	Uterotrophic assay: 0.2–1000 mg/kg/day (PND19-21) Pubertal assay: 200–600 mg/kg/day (PND22-42)	No estrogenic activity in the uterotrophic assay and did not alter puberty in male and female rats or mammary gland development in female rats.	
	GFP-ERα/β:PRL-HeLa cell MCF-7 ERE-MAR cells	HeLa: 50 pM–5 μM TMBPF/-DGE/-ER for 1 or 24 h ± 10 nM E2 MCF-7: 2 or 5 μM TMBPF/-DGE/-ER ±10 nM E2 for 24 h.	TMBPF exhibited anti-estrogenic activity, whereas its derivatives, DGE and ER, showed no estrogenic activity.	Szafran et al., 2017 [16]
	Human CYP19 + P450 reductase Supersomes	10^−4^ to 10^−10^ M for 15 min with the same concentrations of Formestane	No aromatase inhibition observed, conducted according to EPA OCSPP 890.1200.	Maffini et al., 2020 [17]
	H295R cells	0.0001–100 μM for 48 h	Induced estradiol synthesis at near-cytotoxic concentration (10 μM), conducted according to OECD TG 456.	
	CD-1 mice (8 weeks) KGN cells	50 or 200 μg/kg/day from GD7-PND21 0.01–100 μM for 48 h	Increased E2, FSH, and LH and induced ovarian fibrosis in offspring Elevated oxidative stress and triggered ERRB–p21–dependent cellular senescence.	Fan et al., 2025 [23]
Androgenic	2PB-mCherry-NLS:LnCaP cells	50 pM–5 μM TMBPF/-DGE/-ER for 1 or 24 h ± 10 nM DHT	TMBPF exhibited anti-estrogenic and anti-androgenic activity, whereas its derivatives, DGE and ER, showed no estrogenic or androgenic activity.	Szafran et al., 2017 [16]
	Castrated Rats H295R cells	100–1000 mg/kg/day for 10 days 0.0001–100 μM for 48 h	No androgenic activity and weak anti-androgenic activity up to 1000 mg/kg-bw/day, as assessed by the Hershberger assay (OECD TG 441). Weak induction of testosterone, differing from the testosterone reduction observed, as assessed by OECD TG 456 steroidogenesis assay.	Maffini et al., 2020 [17]
	Yeast-based AR reporter assay In silico docking	0.01–10 μM for 48 h Molecular simulation of AR binding	Strong anti-androgenic activity about 57 times higher than BPA, with binding to the androgen receptor at Gln711 and Thr877.	Park et al., 2024 [24]
	*C. elegans*	0.5 mM for 48 h	Reduced sperm size and embryo survival, without decreases in sperm activity or offspring size; overall weaker toxicity than BPA.	Higley et al., 2024 [25,26]
Thyroidal	Zebrafish larvae	0.05–500 μg/L (0.1% DMSO) for 14 days (from 2 hpf)	Elevated T3 and suppressed GH, with reduced growth, hatchability, and survival, and disruption of the HPT and GH/IGF axes.	Kim et al., 2022 [12]
Obesogenic	hASCs	0.01 and 0.1 μM with/without 10 μM E2	Inhibited adipogenesis, with concomitant cytotoxicity and apoptosis induction.	Cohen et al., 2021 [35]
	3T3-L1 cells	0.01–20 μM (0.1% DMSO)	Showed increased cytotoxicity and, in 3T3-L1 cells, promoted lipid accumulation and PPARγ activity.	Singh et al., 2024 [36]
Skeletal	RAW264.7 (osteoclast differentiation)	0.1, 1, and 5 µM for 72 h with RANKL	Enhanced RANKL-induced osteoclast differentiation, with increased TRAP-positive cells, upregulation of *Nfatc1* and *CtsK*, and activation of MAPK signaling.	Kim et al., 2021 [37]
Teratogenic and developmental	Chicken embryo	0.003 to 30 μM (E5-12)	Increased mortality, reduced body size, facial and gastrointestinal malformations, and greater toxicity than BPA (LC_50_ 1.18 µM vs. 2.92 µM).	Harnett et al., 2021 [14]
Developmental neuronal	Zebrafish embryos and larvae	0.25–8 mg/L for 6d (from 4hpf)	Reduced hatch rate, spinal deformities, reduced swimming activity; impaired CNS development with increased ROS; effects reversed by NAC.	Liang et al., 2023 [48]
NIAS-related genotoxicity	LC-TOF-MS Integrated in vitro genotoxicity test battery	Migration test extract of epoxy lining + Ames (OECD TG471), micronucleus (OECD TG487) and Migratox assay	No genotoxicity was detected in validated assays, while epoxide-containing NIAS were identified and prioritized.	Mallen et al., 2023 [53]
Gut microbiota disruption	Human gut microbiota	1 mM for 48 h exposure (anaerobic cultivation)	Less impact on gut microbiota diversity and SCFA production than other bisphenols, while microbial adsorption reduced estrogenicity; meanwhile, the gut microbiota culture supernatant decreased Caco-2 cell viability.	Średnicka et al., 2024 [56]

Abbreviations: NOAEL, no-observed-adverse-effect level; LC_50_, median lethal concentration; TRAP, tartrate-resistant acid phosphatase; Nfatc1, nuclear factor of activated T cells, cytoplasmic 1; Ctsk, cathepsin K; ERRβ, estrogen-related receptor beta; AR-LBD, androgen receptor ligand-binding domain; NIAS, non-intentionally added substances; SCFA, short-chain fatty acids; HPT axis, hypothalamus–pituitary–thyroid axis; GH/IGF axis, growth hormone/insulin-like growth factor axis; CNS, central nervous system; MAPK, mitogen-activated protein kinase; NAC, N-acetylcysteine.

**Table 3:** Comparison of BPA and TMBPF toxicity results within the same study.

Toxicity	Tested Assay and Results (BPA vs. TMBPF)
Estrogenic and anti-estrogenic	Fluorescent reporter array in GFP-ERα/ERβ PRL-HeLa [16] - BPA: Estrogenic activity (5 µM, 1 h/24 h), no anti-estrogenic effect - TMBPF: No estrogenic but clear anti-estrogenic effect - TMBPF derivatives (-DGE/-ER): No effect Integrated ERE-luciferase reporter assay [16] - BPA: Estrogenic effect only (5 µM) - TMBPF: No effect - TMBPF derivatives: Anti-estrogenic effect
Androgenic and anti-androgenic	Integrated probasin-mCherry-NLS reporter assay [16] - No effect with compounds alone - With 5 µM + dihydrotestosterone (DHT): BPA showed androgenic effect (223% of DHT), TMBPF showed anti-androgenic effect (21% of DHT)
Androgenic and anti-androgenic	Yeast-based reporter assay [24] - Agonist: none - Antagonist (IC_50_): BPA 20.6 µM, TMBPF 0.36 µM (~57× potency)
Male fertility	Fertility and embryonic lethality of *C.elegans* [25,26] - Brood size: BPA↓ (*p* < 0.05), TMBPF no effect - Embryonic lethality: Both↑ (*p* < 0.05) - Spermatid size: Both↓ (*p* < 0.05) - Spermatid activation: only BPA↓ (*p* < 0.05)
Cytotoxic	Cytotoxicity in rat adipose-derived stem cell (rASCs) and human ASCs (hASCs) [18] - TMBPF showed cytotoxicity from 0.01 µM, with LC_50_ values of 0.88 µM (rASC) and 0.06 µM (hASC), being ~100× more potent than BP
Obesogenic	Analyze mean ratio of lipid vacuoles to cell number in lipid differentiated: Lipid vacuoles/Cell number [35] - BPA: ↑at 0.1 µM (*p* < 0.01), ↓at 1 µM (*p* < 0.01) - TMBPF: ↓at 0.01–0.1 µM (*p* < 0.01) in hASCs
Obesogenic	Lipid accumulation in differentiating 3T3-L1 cells [36] - 3T3-L1 lipid accumulation: Both ↑from 1 µM - TMBPF > BPA at 10 µM (*p* < 0.001) and 20 µM (*p* < 0.05)
Developmental	Development, body size, tissue morphology, and reproduction of *C.elegans* [66] - BPA: ↓adulthood fraction, length, width, brood size (*p* < 0.01–0.0001) - TMBPF: ↓adulthood fraction, brood size (*p* < 0.05). Neurite beading formation in *C.elegans* axon [48] - BPA: ↑at day 9 adults (*p* < 0.01) - TMBPF: ↑at day 1 adults (*p* < 0.01). Healthspan of *C.elegans* [48] - BPA: ↓body speed, wavelength, survival (*p* < 0.05–0.001) - TMBPF: no effect.
Skeletal	Activation of Osteoclast Differentiation in RAS264.7 cell: Osteoclast area [37] - BPA: ↑at 10 µM (*p* < 0.05) - TMBPF: ↑at 1 µM (*p* < 0.05), 10 µM (*p* < 0.001) Tartrate-resistant acid phosphatase (TRAP) effect [37] - BPA: no effect - TMBPF: ↑at 5 µM (*p* < 0.001).
Teratogenic	Chicken embryo survival (LC_50_) [14] - BPA:2.92 μM, TMBPF: 1.18 μM

Abbreviations: ERα/ERβ, estrogen receptor alpha/beta; PRL-HeLa, prolactin promoter–HeLa cell line; ERE, estrogen response element; IC_50_, half maximal inhibitory concentration; *C. elegans*, *Caenorhabditis elegans*; LC_50_, median lethal concentration; ↑ indicates an increase; ↓ indicates a decrease (vs. control).

## Data Availability

Not applicable.

## Supplementary Materials

The following supporting information can be downloaded at: https://www.mdpi.com/article/10.3390/ijms26199280/s1, References [68,69] are cited in the supplementary materials
