# Supplementary material for: Tetramethyl Bisphenol F: Organ- and System-Specific Toxicity, Current Status, and Perspectives
Source: Int J Mol Sci. 2025 Sep 23;26(19):9280. doi: 10.3390/ijms26199280 (PMC12524657; doi:10.3390/ijms26199280)
Supplement: Supplementary file 1 [file ijms-26-09280-s001.zip › ijms-3843043-supplementary.pdf]

Table S1. Chemical properties of BPA and TMBPF

Property	BPA (Bisphenol A) [68]	TMBPF [69]
Chemical structure	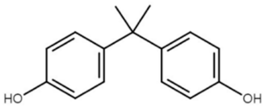	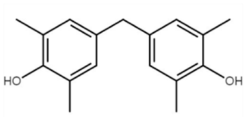
Molecular formula	$C_{15}H_{16}O_2$	$C_{17}H_{20}O_2$
Molecular weight	228.29 g/mol	256.34 g/mol
CAS number	80-05-7	5384-21-4
Melting point	150–157 °C	186 °C
Boiling point	250–252 °C	245 °C
Commercial Introduction	1950's	2017

References

68. Information, N.C.f.B. PubChem Compound Summary for CID 6623, Bisphenol A. Available online: https://pubchem.ncbi.nlm.nih.gov/compound/6623#section=Experimental-Properties (accessed on 30 July 2025).
69. Information, N.C.f.B. PubChem Compound Summary for CID 79345, 4,4'-Methylenebis(2,6-dimethylphenol). Available online: https://pubchem.ncbi.nlm.nih.gov/compound/4_4_-Methylenebis_2_6-dimethylphenol. (accessed on 31 July 2025).
